# A retrospective cross-sectional study of association between triglyceride-glucose index and carotid atherosclerosis

**DOI:** 10.3389/fcvm.2025.1611466

**Published:** 2025-06-06

**Authors:** Congfang Guo, Mingming Li, Lili Wang, Yuting Bai, Yudong Rong, Minying Zhang, Fenghua Guo, Xiang Guo, Jie Guo, Li Zhang, Yiyan Zhao

**Affiliations:** ^1^ Health Management Center, Tianjin First Central Hospital, Tianjin, China; ^2^ Shanxi Bethune Hospital, Shanxi Academy of Medical Sciences, Third Hospital of Shanxi Medical University, Tongji Shanxi Hospital, Taiyuan, China; ^3^ Department of Energy Chemistry and Materials Engineering, Shanxi Institute of Energy, Jinzhong, China; ^4^ School of Medicine, Nankai University, Tianjin, China

**Keywords:** triglyceride-glucose index (TyG index), carotid atherosclerosis (CA), carotid plaque, carotid stenosis, cross-sectional study

## Abstract

**Background:**

The triglyceride-glucose (TyG) index, a simple surrogate marker of insulin resistance (IR), has been associated with cardiovascular risk factors and atherosclerosis. However, its relationship with carotid atherosclerosis (CA), including carotid intima thickening (CMT), plaques, and stenosis, remains inadequately studied in the general population.

**Purpose:**

This study aimed to evaluate the association between the TyG index and CA.

**Methods:**

A retrospective cross-sectional study was conducted among 8,600 participants undergoing health checkups and carotid ultrasonography. CA was defined as the presence of CMT, plaques, or stenosis (>50%). The TyG index was calculated using fasting triglycerides and glucose levels. Multivariate logistic regression and subgroup analyses were performed to assess the association between the TyG index and CA. We constructed fitting curves to evaluate the dose–response relationship between the TyG index and CA in different subgroups. All statistical analyses were executed using R Statistical Software and the Free Statistics analysis platform.

**Results:**

The TyG index was positively associated with CA (OR = 1.22, 95% CI: 1.08–1.38, *P* = 0.003), plaques (OR = 1.28, 95% CI: 1.12–1.47, *P* < 0.001), and stenosis (OR = 2.49, 95% CI: 1.86–3.32, *P* < 0.001), but not with CMT. Subgroup analyses revealed stronger associations in younger individuals (<49 years), males, and those without hypertension, dyslipidemia, or hyperglycemia. A nonlinear relationship between TyG and CA was observed in lean/normal-weight individuals, with a threshold effect at TyG = 8.112. We found that if TyG below 8.112, each unit increasing in TyG reduced CA risk (OR = 0.26, 95% CI: 0.07–0.96, *P* = 0.043), while above it, the risk increased significantly (OR = 1.65, 95% CI: 1.06–2.57, *P* = 0.025) in this study. Linear relationships between TyG index and CA were showed in different subgroups stratified by age, gender and different metabolic conditions and overweight/obesity individals.

**Conclusion:**

The TyG index is a significantly association of CA, particularly in high-risk subgroups. The TyG index shows promise for CA risk stratification. Emphasizing the need for targeted interventions in specific populations ahead of time. The TyG index may complement existing tools, but further prospective validation is needed to assess its incremental value.

## 1 Introduction

Carotid atherosclerosis (CA) is an early pathological marker of atherosclerosis, it often begins in young age and even in childhood, and remains latent and asymptomatic for a long time before entering the advanced stage (1). The main characteristics of CA include CMT, the formation of carotid plaques, and carotid stenosis (2). By 2020, a study estimated that the prevalence of increased carotid intima-media thickness (CIMT) among people aged 30–79 years was 27.65%. Additionally, the prevalence of carotid plaque and carotid stenosis in this population was 21.15% and 1.5% (3). With the development of society, the incidence of metabolic diseases is increasing. The study found that CIMT is elevated, particularly in individuals with metabolic conditions such as diabetes (4). Another study showed that the greater the increase in CIMT values, the higher the risk of stroke (5). Additionally, CIMT measurement helps to assess the risk and incidence of cardiovascular disease (6). These findings highlight the critical role of CA evaluation in early cardio-cerebro vascular disease screening, particularly for identifying and intervening in high-risk populations.

Insulin resistance (IR) is a core pathological mechanism of metabolic diseases (e.g., obesity, type 2 diabetes, and metabolic syndrome) and an important risk factor for atherosclerosis. However, traditional methods for assessing IR, such as the hyperinsulinemic-euglycemic clamp test, are complex and costly, limiting their application in large-scale population studies. The triglyceride-glucose index (TyG index), a simple indicator based on fasting triglycerides and fasting glucose, has recently been widely used to evaluate IR (7). Studies have shown that the TyG index not only has a strong predictive ability for insulin resistance but is also closely associated with various cardiovascular risk factors and the development of atherosclerosis (8).

However, only a few studies have focused on the relationship between the TyG index and CA among the general population, and the results have been inconsistent. Irace C et al. demonstrated an association between the TyG index and CA in a cohort of 1,432 subjects (9). However, the study did not include detailed analyses, such as stratification by different CA types or subgroup evaluations. Wenzhen Li et al. conducted a study involving 59,123 patients, which demonstrated an association between the TyG index and CA (10). However, the study population was limited to individuals aged 40 years and older.

Our study focuses on adults aged 18 years and older, aiming to systematically analyze the association between the TyG index and different phenotypes of CA (CMT, plaques, and stenosis). By incorporating cross-sectional data, this research will provide more generalizable evidence for risk of CA based on the TyG index.

## 2 Participants and methods

### 2.1 Study population and ethics

**Figure 1:**
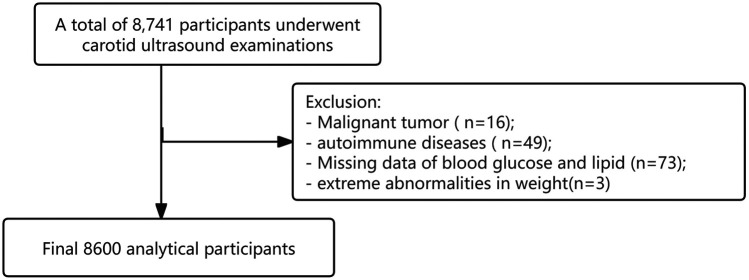
Study participants flow chart.

In this retrospective study, individuals who underwent annual health checkups along with carotid ultrasound examinations were identified by reviewing case notes using electronic medical records at Tianjin First Central Hospital from November 2023 to November 2024. 8,741 individuals were included. Individuals were made up of Han Chinese individuals and excluded if they were younger than 18 years, previously diagnosed with severe malignant tumor, autoimmune diseases and transplantation of various organs and so on requiring long-term use of hormones and immunosuppressants. Because pregnancy can significantly alter metabolic and physiological parameters, including blood glucose and lipid levels, which may skew the results and complicate the interpretation of the relationship between the TyG index and CA. Those who were pregnant were also excluded. 16 participants of malignant tumor and 49 participants of autoimmune diseases were excluded. Individual records were anonymized and de-identified prior to the analysis. During the analysis, 73 participants were excluded due to missing blood glucose and lipid data, and 3 participants were excluded due to abnormal weight data. Ultimately, 8,600 participants were included in our study (Figure 1). According to Kendall's rough sample size estimation rule, the sample size should be 5–10 times the number of research variables. Considering the validity of the sample, we expanded the overall sample by 10%–20%, resulting in an estimated sample size of approximately 2,000 participants. Our study ultimately included 8,600 participants, which far exceeds the calculated sample size. Approval for the study was granted by the Medical Ethics Committee of Tianjin First Central Hospital (KYAP2025-29).

### 2.2 Definition of carotid atherosclerosis

Two proficient ultrasound technologists, who were unaware of the patient's clinical information, performed carotid ultrasonography examinations in a skilled and autonomous manner. The participants' bilateral carotid arteries were scanned in the supine position with the neck in a hyperextended position. An increased CIMT was defined as a range of 1.0–1.5 mm, which is in line with previous research (10). Carotid plaques were identified as having an intima-media thickness exceeding 1.5 mm or protruding into the lumen by 50% more than the surrounding intima-media thickness (10). Carotid stenosis was defined as the occlusion or more than 50% stenosis of at least one common carotid or internal carotid artery (10). Participants exhibiting CMT, plaques, or carotid stenosis were diagnosed with CA.

### 2.3 Assessment of covariates

A face-to-face interview with a simple questionnaire was conducted to collect the participant's demographical characteristics, disease history, surgical history and medication history. The previous coronary angiography indicated vascular stenosis, suggesting a history of coronary heart disease. Well-trained nurses conducted anthropometric measurements of height, weight, and seated blood pressure following international standards. Body weight and standing height were measured using Ultrasonic Height and Weight Measuring Instrument SG-1001SA (Beijing Chioy Medical technology Co., Ltd, Beijing, China). It was calibrated regularly to ensure accuracy. Seated blood pressure was measured with a fully automatic electronic sphygmomanometer ABP-1000SA (Beijing Chioy Medical technology Co., Ltd, China). Body mass index (BMI) was calculated and overweight was defined as BMI ≥ 23 kg/m^2^, according to the suggestions of the WHO for Chinese populations (11).

After 12 h of fasting at night, venous blood samples were collected early in the morning for biochemical analysis in the certified laboratory at Tianjin First Central Hospital. Biochemical auto analyzer was employed for measuring fasting plasma glucose (FBG), lipid profiles, liver enzymes, creatinine, urea nitrogen and uric acid levels. Glycated hemoglobin A1c (HbA1c) levels were measured using high-performance liquid chromatography. TyG was calculated as the natural logarithm (Ln) of [TG (mg/dl) × FBG (mg/dl)/2] (12).

### 2.4 Definitions of metabolic diseases

Hyperglycemia was defined as FPG ≥ 7.0 mmol/L or HbA1c ≥ 6.5% or previously diagnosed as diabetes (13). Hypertension was defined as systolic blood pressure (SBP) ≥140 mmHg and/or diastolic blood pressure (DBP) ≥90 mmHg or pre-existing hypertension. Dyslipidemia was identified as triglyceride (TG) ≥2.3 mmol/L or total cholesterol (TC) ≥6.2 mmol/L or low-density lipoprotein cholesterol (LDL-C) ≥4.1 mmol/L or high-density lipoprotein cholesterol (HDL-C) <1.0 mmol/L, in accordance with the Chinese guidelines for the management of dyslipidemia in adults (2016) (14) or prior diagnosis of dyslipidemia. Diagnosis of metabolic dysfunction-associated fatty liver disease (MAFLD) was based on the new definition (15).

### 2.5 Statistical analysis

All normally distributed continuous variables were expressed presented as mean ± SD, and skewed continuous variables were described as median (interquartile range [IQR]). Categorical variables were presented as frequencies (%). Multinomial logistic regression models were employed to assess the associations between variable X and outcome Y. Variable X was treated as a categorical variable, divided into tertiles. The selection of potential confounders was guided by clinical judgment and existing scientific literature. We developed a total of three models to evaluate these associations: Model 1: This model served as the unadjusted reference. Model 2: This model included adjustments for gender and age. Model 3: In addition to the adjustments made in Model 2, this model further accounted for BMI, smoking status, and a history of hypertension, diabetes, and coronary heart disease, as well as SBP, DBP, LDL, HDL and TC.

We employed a restricted cubic spline model to develop smooth curves that facilitate the examination of potential nonlinear dose-response relationships between variable X and outcome Y. In this model, X was treated as a continuous variable, utilizing four knots at the 5th, 35th, 65th, and 95th percentiles. The presence of non-linearity was assessed using a likelihood ratio test. Shaded areas represent 95% confidence intervals. Upon identifying a non-linear association, a two-piecewise regression analysis was conducted to determine the threshold effect of X on Y, informed by the smoothing plot. Additionally, subgroup analyses were performed to explore the relationship between TyG and CA across various subgroup variables. Missing data, which constituted less than 10% of the dataset, were addressed using listwise deletion on an analysis basis.

All statistical analyses were executed using R Statistical Software (Version 4.2.2, http://www.R-project.org, The R Foundation) and the Free Statistics analysis platform (Version 1.9, Beijing, China, http://www.clinicalscientists.cn/freestatistics). A two-sided *p*-value of less than 0.05 was deemed statistically significant.

## 3 Results

### 3.1 Demographic and clinical characteristics of the study population in each of TyG index tertiles

The mean age was 49.5 ± 11.9 years and 69.8% were male. The prevalence of CA was 38.9%, including 9.3% of CMT, 28.6% of carotid plaque and 1% of carotid stenosis in the total population. Table 1 presents the demographic and clinical characteristics of the participants according to TyG index tertiles. The mean age was slightly higher in T3 (50.6 ± 10.9 years) compared to T1 (47.0 ± 12.5 years). The proportion of males increased with the TyG index, being 54.6% in T1, 72.6% in T2, and 82.2% in T3. However, the proportion of females decreased with the TyG index, being 45.4% in T1, 27.4% in T2, and 17.8% in T3. Smoking and drinking rates significantly increased in groups with higher TyG index. For example, smoking rates rose from 13.4% in T1–28.5% in T3, and drinking rates increased from 4.4%–10.2%. Hepatic and renal function parameters, including aspartate aminotransferase (AST), alanine aminotransferase (ALT), blood urea nitrogen (BUN) and serum creatinine (Cr) levels, were significantly higher in T3 than in T1 or T2.

**Table 1:** Characteristics of participants in the study.

Covariates	Tertiles of TyG index					
	Total (*n* = 8,600)	T1 (*n* = 2,867)	T2 (*n* = 2,866)	*P* value	T3 (*n* = 2,867)	*P* value
Demographic						
Gender, male, *n* (%)	6,002 (69.8)	1,564 (54.6)	2,082 (72.6)	<0.001**	2,356 (82.2)	<0.001**
Age, years	49.5 ± 11.9	47.0 ± 12.5	50.9 ± 11.8	<0.001**	50.6 ± 10.9	0.247
**Smoking**, *n* (%)	1825 (21.2)	383 (13.4)	624 (21.8)	<0.001**	818 (28.5)	<0.001**
**Drinking**, *n* (%)	611 (7.1)	126 (4.4)	193 (6.7)	<0.001**	292 (10.2)	<0.001**
History of disease						
Hypertension, *n* (%)	1,973 (22.9)	373 (13)	657 (22.9)	<0.001**	943 (32.9)	<0.001**
Diabetic, *n* (%)	735 (8.5)	87 (3)	192 (6.7)	<0.001**	456 (15.9)	<0.001**
Heart disease, *n* (%)	301 (3.5)	71 (2.5)	99 (3.5)	0.025*	131 (4.6)	0.038*
Anthropometric characteristics						
Body Mass Index^a^, kg/m^2^	25.9 ± 3.7	24.1 ± 3.4	26.1 ± 3.3	<0.001**	27.5 ± 3.6	<0.001**
SBP^b^, mmHg	130.3 ± 17.7	124.3 ± 16.5	131.1 ± 17.2	<0.001**	135.4 ± 17.7	<0.001**
DBP^b^, mmHg	79.4 ± 11.5	75.0 ± 10.9	79.8 ± 10.9	<0.001**	83.4 ± 11.3	<0.001**
Metabolism-related parameter						
FPG, mmol/L	5.3 ± 1.4	4.7 ± 0.5	5.0 ± 0.8	<0.001**	6.0 ± 2.0	<0.001**
HbA1c^c^, %^c^	5.6 ± 1.0	5.2 ± 0.5	5.4 ± 0.7	<0.001**	6.0 ± 1.3	<0.001**
TC, mmol/L	4.9 ± 1.0	4.6 ± 0.9	4.9 ± 0.9	<0.001**	5.2 ± 1.1	<0.001**
LDL-C, mmol/L	3.0 ± 0.8	2.8 ± 0.7	3.1 ± 0.8	<0.001**	3.1 ± 0.9	0.511
TG, mmol/L	1.4 (1.0, 2.1)	0.9 (0.7, 1.0)	1.4 (1.3, 1.6)	<0.001**	2.5 (2.1, 3.2)	<0.001**
HDL-C, mmol/L	1.2 ± 0.3	1.5 ± 0.3	1.2 ± 0.3	<0.001**	1.0 ± 0.2	<0.001**
UA, umol/L	340.7 ± 86.3	305.6 ± 76.5	344.8 ± 81.7	<0.001**	371.6 ± 87.4	<0.001**
Hepatic and renal condition						
ALT, U/L	19.6 (13.9, 29.1)	15.4 (11.8, 21.6)	19.8 (14.5, 28.7)	<0.001**	24.6 (17.4, 36.7)	<0.001**
AST, U/L	18.5 (15.1, 23.6)	16.9 (14.1, 20.7)	18.8 (15.4, 23.5)	<0.001**	20.1 (16.3, 26.9)	<0.001**
BUN, mmol/L	5.0 ± 1.3	4.9 ± 1.2	5.0 ± 1.3	<0.001**	5.1 ± 1.3	0.03*
Cr, umol/L	74.2 ± 15.8	70.8 ± 14.4	75.4 ± 16.0	0.003**	76.4 ± 16.2	0.01*
CA	3,344 (38.9)	828 (28.9)	1,201 (41.9)	<0.001**	1,315 (45.9)	0.002**
CMT	798 (9.3)	229 (8)	302 (10.5)		267 (9.3)	
Plaques	2,459 (28.6)	588 (20.5)	874 (30.5)		997 (34.8)	
Stenosis	87 (1.0)	11 (0.4)	25 (0.9)		51 (1.8)	
BMI stratification, *n* (%)				<0.001**		<0.001**
Lean/normal^a^	1,715 (19.9)	1,090 (38)	427 (14.9)		198 (6.9)	
Overweight/Obesity	6,884 (80.1)	1,777 (62)	2,438 (85.1)		2,669 (93.1)	
Metabolic disorder						
MAFLD, *n* (%)	4,603 (53.5)	789 (27.5)	1,589 (55.4)	<0.001**	2,225 (77.6)	<0.001**
Hypertension, *n* (%)^b^	3,558 (41.4)	731 (25.5)	1,210 (42.2)	<0.001**	1,617 (56.4)	<0.001**
Hyperglycemia, *n* (%)^c^	1,136 (14.2)	99 (3.8)	253 (9.7)	<0.001**	784 (28.1)	<0.001**
Dyslipidemia, *n* (%)	3,460 (40.2)	354 (12.3)	854 (29.8)	<0.001**	2,252 (78.5)	<0.001**
Hyperuricemia, *n* (%)	1,487 (17.3)	221 (7.7)	481 (16.8)	<0.001**	785 (27.4)	<0.001**

*p*-values were expressed for inter-tertile comparisons (T1 vs. T2 and T2 vs. T3).

^a^ Data missing for 1 patient.

^b^ Data missing for 10 patients.

^c^ Data missing for 593 patients.

*p* values annotated with ‘^*^’ indicate differences (*p* < 0.05). *p* values annotated with ‘^**^’ indicate significant differences (*p* < 0.01).

Participants of the highest tertile (T3) exhibited higher prevalence of history of hypertension, diabetes and coronary heart disease compared to the lowest tertile (T1) and the second tertile (T2). Furthermore, participants of the highest tertile (T3) exhibited higher prevalence of hyperglycemia, hypertension, dyslipidemia, hyperuricemia, MAFLD, and overweight/obesity compared to the lowest tertile (T1) and the second tertile (T2). Moreover, as shown in Table 1, FPG, HbA1c, TG, TC, LDL-C, UA levels were significantly higher in T3 compared to T1 or T2, while HDL-C level of T1 was significantly higher than that of T2 or T3. BMI, SBP and DBP levels of T3 were significantly higher than those of T1 or T2.

The comparison of carotid atherosclerosis among TyG index tertiles showed that carotid atherosclerosis, carotid plaque and carotid stenosis in T3 were significantly higher than those in T1 or T2.

### 3.2 Association of TyG index with CA, CMT, plaques and stenosis

The results of multivariate logistic regression analysis were shown in Table 2. When the TyG index was used as a continuous variable, it was significantly associated with the risk of CA (OR = 1.22, 95% CI: 1.08–1.38, *P* = 0.003), plaques (OR = 1.28, 95% CI: 1.12–1.47, *P* *<* *0.001*) and stenosis severity (>50%) (OR = 2.49, 95% CI: 1.86–3.32, *P* *<* *0.001*). Compared with the T1 in the tertiles of TyG index, T3 was signifcantly associated with a higher risk of CA (OR = 1.3, 95% CI: 1.1–1.54, *P* = 0.002), plaques (OR = 1.43, 95% CI: 1.19–1.72, *P* *<* *0.001*) and stenosis (>50%) (OR = 3.09, 95% CI: 1.51–6.33, *P* *<* *0.001*). However, there were no significant association after full adjustment with the association between TyG index as continuous variable or tertile variable and CMT.

**Table 2:** Odds ratios and 95% CIs for the association of the TyG index with CA, CMT, plaques and stenosis.

TyG			Tertiles of TyG index				
(*n* = 8,600)			T1 (*n* = 2867)	T2 (*n* = 2,866)		T3 (*n* = 2,867)	
CA	OR (95% CI)	*P*	Reference	OR (95% CI)	*P*	OR (95% CI)	*P*
Model 1^a^	1.52 (1.42–1.63)	<0.001	1.00	1.78 (1.59–1.98)	<0.001	2.09 (1.87–2.33)	<0.001
Model 2^b^	1.37 (1.26–1.48)	<0.001	1.00	1.32 (1.15–1.51)	<0.001	1.66 (1.46–1.9)	<0.001
Model 3^c^	1.22 (1.08–1.38)	0.003	1.00	1.17 (1.01–1.35)	0.033	1.3 (1.1–1.54)	0.002
CMT							
Model 1^a^	1.31(1.16–1.46)	<0.001	1.00	1.62(1.34–1.94)	<0.001	1.53(1.27–1.85)	<0.001
Model 2^b^	1.13(0.99–1.28)	0.066	1.00	1.24(1.02–1.51)	0.028	1.22(1–1.49)	0.046
Model 3^c^	1.03(0.65–1.25)	0.75	1.00	1.11(0.9–1.37)	0.312	1.02(0.79–1.31)	0.899
Plaques							
Model 1^a^	1.56(1.45–1.68)	<0.001	1.00	1.82(1.61–2.06)	<0.001	2.23(1.97–2.52)	<0.001
Model 2^b^	1.44(1.31–1.58)	<0.001	1.00	1.35(1.16–1.56)	<0.001	1.83(1.58–2.12)	<0.001
Model 3^c^	1.28(1.12–1.47)	<0.001	1.00	1.2(1.02–1.41)	0.027	1.43(1.19–1.72)	<0.001
Stenosis							
Model 1^a^	2.86(2.23–3.66)	<0.001	1.00	2.78(1.37–5.67)	0.005	6.09(3.16–11.73)	<0.001
Model 2^b^	3.27(2.46–4.34)	<0.001	1.00	2.18(1.06–4.48)	0.033	5.43(2.79–10.57)	<0.001
Model 3^c^	2.49 (1.86–3.32)	<0.001	1.00	2.07 (1.01–4.25)	0.047	3.09 (1.51–6.33)	<0.001

^a^ Model 1 was crude model.

^b^ Model 2 was adjusted for the variables in model 1 plus gender and age.

^c^ Model 3 was adjusted for the variables in Model 2 plus BMI, smoking, history of hypertension, diabetes, coronary heart disease, SBP, DBP, LDL, HDL, TC.

### 3.3 Subgroup analyses for the relevance of TyG index with CA, CMT, plaques and stenosis

**Figure 2:**
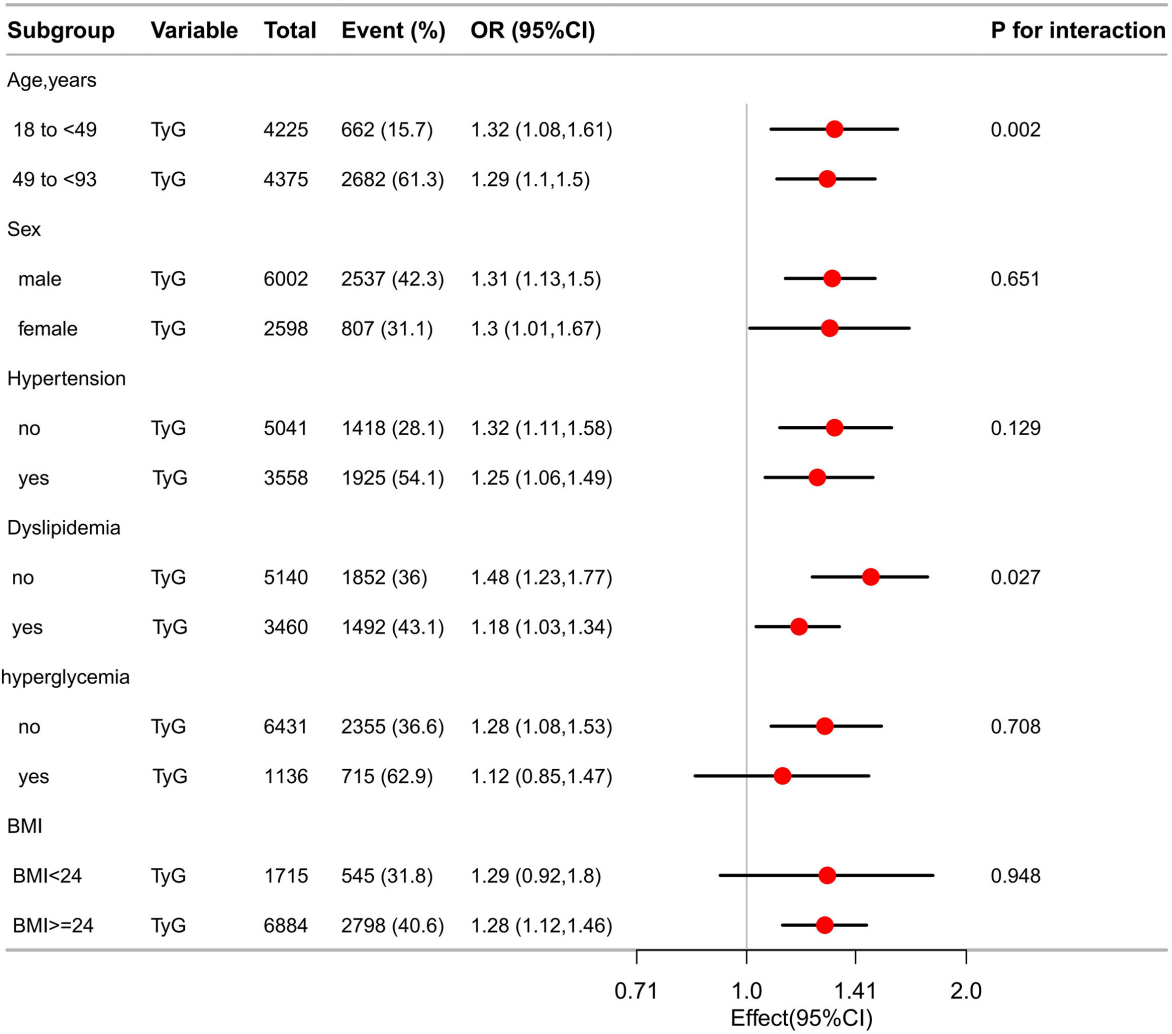
Association of TyG with CA among 8,600 participants in different patient subgroups.

The TyG index demonstrated a consistent and positive correlation with the odds of CA, plaques, and stenosis across various subgroups stratified by age, sex, presence or absence of hypertension, dyslipidemia, hyperglycemia, and BMI (Table 3). Subgroup analysis revealed that OR for the association between the TyG index and CA were higher among individuals younger than 49 years compared to those aged 49 years or older (OR = 1.32, 95% CI: 1.08–1.61, *P* = 0.006 vs. OR = 1.29, 95% CI: 1.1–1.5, *P* = 0.001), men compared to women (OR = 1.31, 95% CI: 1.13–1.5, *P* < 0.001 vs. OR = 1.3, 95% CI: 1.01–1.67, *P* *=* *0.042*), individuals without hypertension compared to those with hypertension(OR = 1.32, 95% CI: 1.11–1.58, *P* = 0.002 vs. OR = 1.25, 95% CI: 1.06–1.49, *P* = 0.009), individuals without dyslipidemia compared to those with dyslipidemia(OR = 1.48, 95% CI: 1.23–1.77, *P* *<* *0.001* vs. OR = 1.18, 95% CI: 1.03–1.34, *P* = 0.013) (Figure 2). The association between TyG and CA remained in non-hyperglycemic (OR = 1.27, 95% CI: 1.08–1.5, *P* = 0.006) and overweight/obese people (OR = 1.28, 95% CI: 1.12–1.46, *P* < 0.001), but not in hyperglycemic (OR = 1.12, 95% CI: 0.85–1.47, *P* = 0.414) and normal/lean weight people (OR = 1.29, 95% CI: 0.92–1.8, *P* = 0.138) (Figure 2).

**Table 3:** Subgroup analyses for the association of the continuous TyG index with CA, CMT, plaques and stenosis.

Subgroup	Total	Event %	OR, 95% CI			
			CA	CMT	Plaque	Stenosis
Age						
18–<49	4,225	662 (15.7)	1.32 (1.08, 1.61)	1.04 (0.75, 1.45)	1.49 (1.18, 1.88)	1.27 (0.55, 2.95)
49–<93	4,375	2,682 (61.3)	1.29 (1.1, 1.5)	1.05 (0.83, 1.32)	1.33 (1.13, 1.57)	2.9 (2.21, 3.82)
Sex						
Male	6,002	2,537 (42.3)	1.31 (1.13, 1.5)	1.06 (0.86, 1.32)	1.38 (1.19, 1.61)	3.17 (2.23, 4.5)
Female	2,598	807 (31.1)	1.3 (1.01, 1.67)	0.95 (0.63, 1.43)	1.44 (1.09. 1.89)	2.26 (1.37, 3.71)
Hypertention						
No	5,041	1,418 (28.1)	1.32 (1.11, 1.58)	1.07 (0.82, 1.41)	1.43 (1.18, 1.75)	2.52 (1.42, 4.47)
Yes	3,558	1,925 (54.1)	1.25 (1.06, 1.49)	1.02 (0.79, 1.31)	1.31 (1.09, 1.57)	2.68 (1.62, 4.46)
Dyslipidemia						
No	5,140	1,852 (36)	1.48 (1.23, 1.77)	1.33 (1.02, 1.73)	1.51 (1.24, 1.85)	6.21 (3.97, 9.71)
Yes	3,460	1,492 (43.1)	1.18 (1.03, 1.34)	0.93 (0.76, 1.14)	1.24 (1.08, 1.43)	2.36 (1.76, 3.16)
Hyperglycemia						
No	6,871	2,576 (37.5)	1.27 (1.08, 1.5)	1.07 (0.83, 1.36)	1.35 (1.14, 1.62)	2.06 (1.4, 3.04)
Yes	1,136	715 (62.9)	1.12 (0.85, 1.47)	0.99 (0.62, 1.58)	1.12 (0.84, 1.49)	3.06 (1.92, 4.88)
BMI						
<24	1,715	545 (31.8)	1.29 (0.92, 1.8)	0.79 (0.46, 1.35)	1.51 (0.96, 2.16)	3.4 (1.49, 7.78)
>=24	6,884	2,798 (40.6)	1.28 (1.12, 1.46)	1.07 (0.88, 1.31)	1.35 (1.17, 1.55)	2.73 (2.12, 3.53)

**Figure 3:**
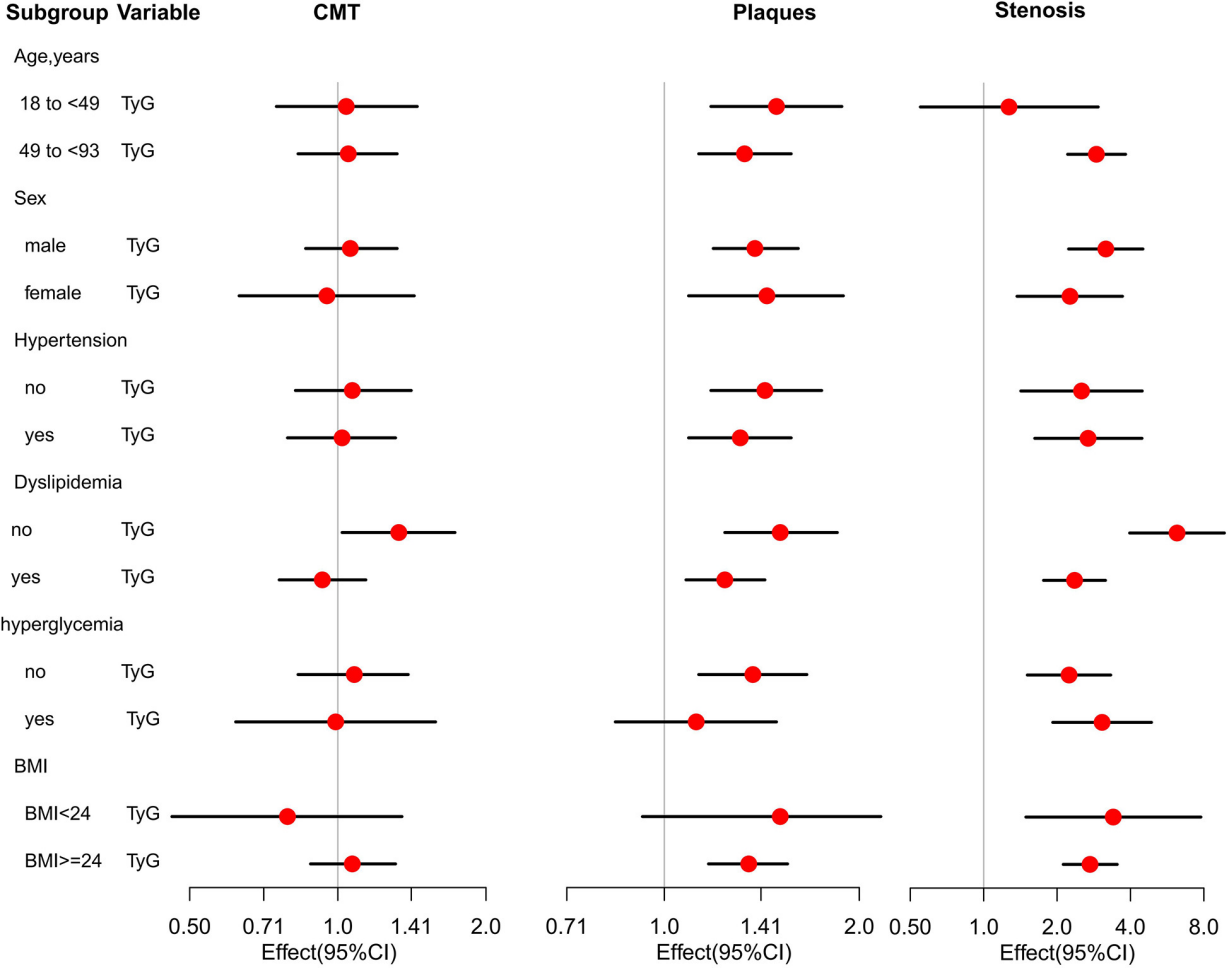
Association of TyG with CMT, plaques and stenosis among 8,600 participants in different patient subgroups.

As showed in Figure 3, we also found that no significant association was observed between the TyG index and CMT across subgroups defined by age, sex, presence or absence of hypertension, hyperglycemia, or BMI. Notably, in the subgroup without dyslipidemia, the TyG index was associated with CMT (OR: 1.33, 95% CI: 1.02–1.73, *P* = 0.041).

As showed in Figure 3, the results showed that more significant association with plaques (OR = 1.49, 95% CI: 1.18–1.88, *P* = 0.011) in younger group (18–48years) than in older group and there was still association between TyG and stenosis risk (OR = 2.9, 95% CI: 2.21–3.82, *P* *<* *0.001*) in older group (49–93years), but no association in younger group (OR = 1.27, 95% CI: 0.55–2.95, *P* = 0.478). We also found that there was a higher risk of plaques among females (OR = 1.44, 95% CI: 1.09–1.89, *P* = 0.005) than males while a higher risk of stenosis in males (OR = 3.17, 95% CI: 2.23–4.5, *P* *<* *0.001*) than females (OR = 2.26, 95% CI: 1.37–3.71, *P* *<* *0.001*).The findings showed that higher risk of plaque (OR = 1.43, 95% CI: 1.18–1.75, *P* = 0.006) and lower risk of stenosis (OR = 2.52, 95% CI: 1.42–4.47, *P* = 0.001) in without hypertension participants than the hypertension participants (OR = 1.31, 95% CI: 1.09–1.57, *P* = 0.04 and OR = 2.68, 95% CI: 1.62–4.46, *P* = 0.001). The study also showed that higher risk of plaque (OR = 1.51, 95% CI: 1.24–1.85, *P* = 0.001) and extreme stenosis risk (OR = 6.21, 95% CI: 3.97–9.71, *P* = 0.001) in normal lipid group than dyslipidemia group (OR = 1.24, 95% CI: 1.08–1.43, *P* = 0.033 and OR = 2.36, 95% CI: 1.76–3.16, *P* = 0.006). The findings showed that high risk of plaque (OR = 1.35, 95% CI: 1.14–1.62, *P* = 0.001) in non-hyperglycemia participants, but no relation with hyperglycemia participants (OR = 1.12, 95% CI: 0.84–1.49, *P* = 0.317). There was lower risk of stenosis (OR = 2.06, 95% CI: 1.4–3.04, *P* = 0.043) in non-hyperglycemia participants than the hyperglycemia participants (OR = 3.06, 95% CI: 1.92–4.88, *P* = 0.015). We also found that there were high risk of plaque (OR = 1.35, 1.17–1.55, *P* = 0.002) in overweight/obesity population, but no relation in lean/normal weight population (OR = 1.51, 0.96–2.16, *P* = 0.057). There were high stenosis risk (OR = 3.4, 95% CI: 1.49–7.78, *P* = 0.029 vs. OR = 2.73, 95% CI: 2.12–3.53, *P* = 0.007) in lean/normal weight population than in overweight/obesity population.

### 3.4 A fitting curve between the TyG index level and CA

**Figure 4:**
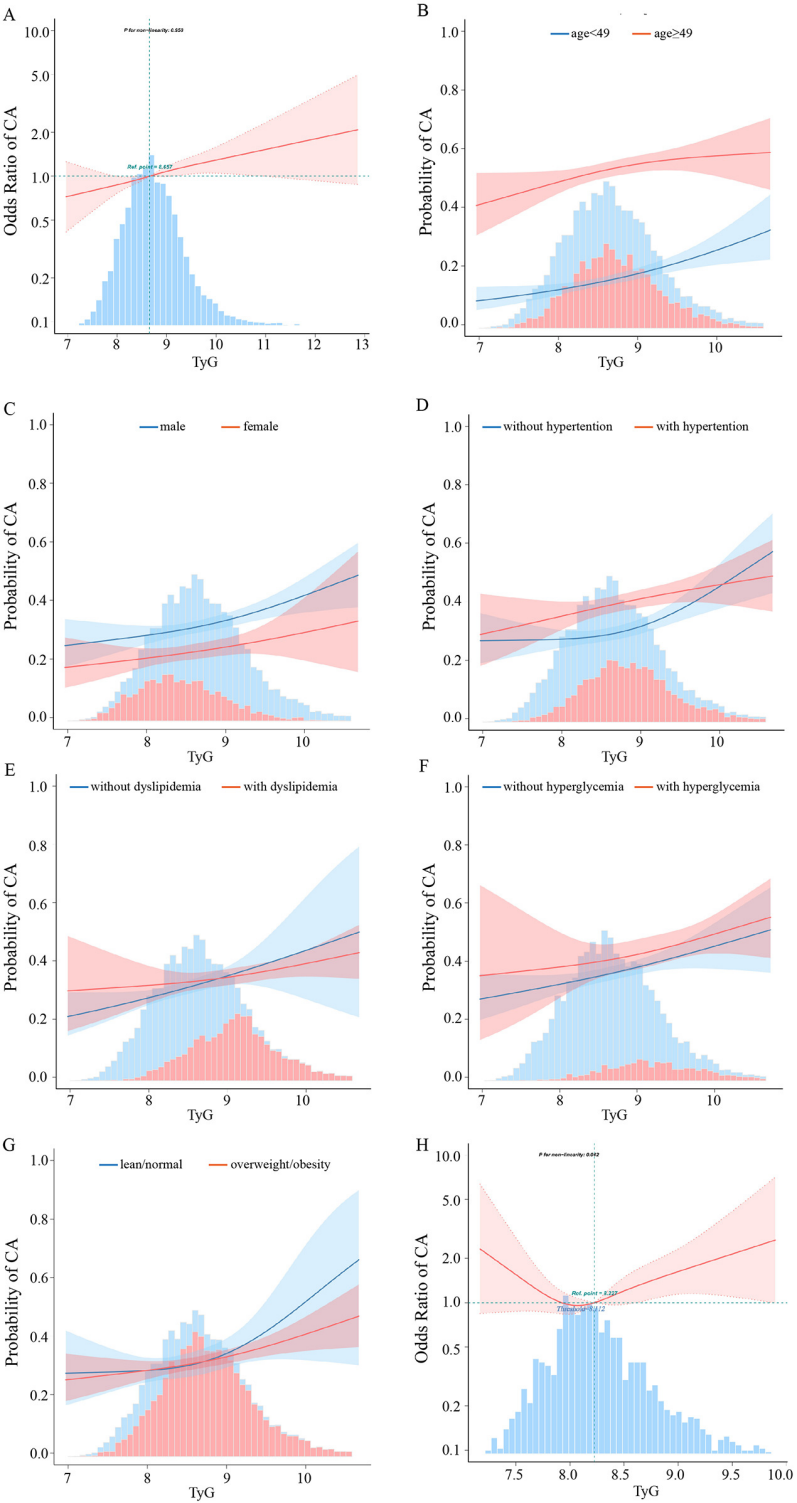
The relationship between the TyG index and CA. **(A)** Relationship in the total participants. **(B)** Relationship in the younger than 49 years participants and 49 years or older participants, respectively. **(C)** Relationship in the male and female participants. **(D)** Relationship in the without and with hypertension participants, respectively. **(E)** Relationship in the without and with dyslipidemia participants, respectively. **(F)** Relationship in the without and with hyperglycemia participants, respectively. **(G)** Relationship in the lean/normal and overweight/obesity participants, respectively. **(H)** Relationship in the lean/normal weight participants. Only 99% of the data is displayed. Odds ratios are indicated by solid lines and 95% CIs by shaded areas.

To evaluate the dose–response relationship between the TyG index and CA after adjusted for covariates, we constructed fitting curves as Figure 4. As shown in the Figure 4A, there was a positive linear relationship between the TyG index and CA. Figure 4B showed that individuals aged ≥49 years exhibited a stronger probability of CA with the same TyG index in the linear association compared to those aged <49 years. Figure 4C showed that the TyG-CA association was linear in males and in females, with more probability of CA in males than in females. Figures 4D–F showed that participants with hypertension/hyperglycemia/dyslipidemia exhibited a stronger probability of CA with the same TyG in the linear association compared to those without hypertension/hyperglycemia/dyslipidemia. Figures 4G, H illustrated that the risk of CA significantly increased as the TyG index rose in both groups, but the growth curves showed linear in overweight/obesity participants and nonlinear in lean/normal weight participants.

### 3.5 Threshold effect analysis of TyG index on CA in lean/normal weight participants

Table 4 showed that a significant threshold effect of TyG on CA was observed at TyG = 8.112 (*P* for nonlinearity = 0.042) in lean/normal weight individuals. Below this threshold, each unit increase in TyG was associated with a 74% reduced CA risk (OR = 0.26, 95% CI: 0.07–0.96, *P* = 0.043), whereas above the threshold, the risk increased by 65% per unit (OR = 1.65, 95% CI: 1.06–2.57, *P* = 0.025).

**Table 4:** Threshold effect analysis of TyG index on CA in lean/normal weight participants.

Threshold of TyG index	OR	95%	*P-*value
<8.112	0.26	0.07–0.96	0.043
≧8.112	1.65	1.06–2.57	0.025
Non-linear test			0.042

## 4 Discussion

It is well-known that CA is an independent risk factor for stroke and coronary heart disease (16). Increased CIMT was associated with main CVD (Cardiovascular Disease)-related risk factors (17). Approximately 30% of ischemic strokes are attributed to the presence of carotid plaques (18). Thus, identifying credible risk factors and reducing the incidence of CA is essential for the early prevention of acute cardio cerebrovascular diseases. IR plays a crucial role in the development and progression of CA. However, assessment methods for IR, such as the hyperinsulin-normoglycemic clamp technique and the homeostasis model assessment of insulin resistance (HOMA-IR), are often limited in large-scale clinical studies due to their high costs (19). In contrast, TyG index has emerged as a reliable and cost-effective marker of IR. Its advantages include ease of measurement and accessibility (7). Notably, the TyG index (AUC: 0.640) exhibits superior predictive capability for the prevalence of type 2 diabetes compared to HOMA-IR (20). TyG serve as an effective alternative biomarker for IR and has gained increasing attention due to its ability to evaluate arterial stiffness and the risk of cardiovascular and cerebrovascular diseases (21). There have been studies on the association between TyG and CA, primarily focusing on populations aged over 40 or elderly individuals, but the correlation in younger populations remains to be further explored. Our study is the first to provide evidence of a positive association between the TyG index and the risk of CA, including plaques and stenosis, in individuals aged 18 years and older in Tianjin. Furthermore, our study evaluated these associations stratified by sex, age, and the presence or absence of hypertension, hyperglycemia, dyslipidemia, and BMI. The findings were consistent across various clinical subgroups, underscoring their robustness.

Studies have shown that LDL as well as glycated hemoglobin (HbA1c) are significantly correlated with carotid atherosclerosis (22). In our receiver operating characteristic (ROC) curve analysis with CA, the areas under the curve (AUC) for HbA1c and LDL were 0.607 and 0.541, respectively. The AUC for TyG was 0.663, which is higher than its discriminative ability for LDL and HbA1c (Supplementary Figures S1, S2). In our study of 8,600 participants (mean age: 49.5 ± 11.9 years; 69.8% male), we observed a positive correlation between the TyG index and CA including plaque and stenosis, but no significant association with CIMT. Data from the Stroke Screening and Prevention Project (Hubei Province, China, 2017–2020) demonstrated a significant association between the TyG index and CA prevalence, carotid plaques, stenosis severity, and CMT[14]. However, a prior study with 2,830 participants found no significant link between the TyG index and CMT or carotid plaques (23). The discrepancy may stem from differences in sample size and population characteristics. For example, our study and Wenzhen Li *et al*.'s study (10) all included larger, more diverse populations, enhancing statistical power, compared to relatively small sample size of Zhao S *et al*.'s study (23). Wenzhen Li *et al*.'s study (10) focused on individuals above 40 years (mean age 60.03 ± 10.75years) and Zhao S *et al*.'s study focused on elderly individals (mean age 71.5 ± 6.2years). The analysis of early subclinical atherosclerosis progression has shown that atherosclerosis typically occurs in young and middle-aged individuals (24). Our study found that 7.1% of participants under the age of 40 years were examined to have CA (Supplementary Table S1). Therefore, research in younger participants is crucial, too. Populations in our study included adults aged 18 years and above. Additionally, variations in gender and metabolic disorder prevalence across populations may influence the different relationship.

It is well konown that CA is a chronic, progressive pathological process resulting from the interplay of various complex factors at different stages, which progressively advances with age. Subgroup analysis showed that TyG index was significantly related to stenosis in older individuals Moreover, a greater OR for CA, including plaques, was found in individuals under 49 years, which is consistent with previous study (10). This suggests that increased attention should be paid to young people, too. Additionally, the association between TyG and CA including plaques and stenosis, all remained in men and women. A higher OR of plaques was observed in females which is consistent with Ya-Ke Lu et al's study (25). It has been reported that men have more circulating CD14+ and CD16++ monocytes, which have been associated with impaired endothelial function, intima-media thickness, and less carotid compliance, than women (26). These underlying mechanisms may weaken the correlation between the TyG index and carotid artery plaques in males.

Wu Z et al. found that TyG index was also significantly associated with the progression of arterial stiffness in hypertensive people (21). Another study showed that the TyG index could be used to predict the extent of carotid artery disease equally well regardless of hyperlipidaemia (27). Our study also found that the association between the TyG index and CA including plaques and stenosis remained regardless of hypertension and dyslipidemia or not. Furthermore, a higher OR for CA, including plaques was found in individuals without hypertension or dyslipidemia compared to those with hypertension or dyslipidemia The possible explanation is that in individuals without hypertension or dyslipidemia, an elevated TyG index predominantly reflects the direct impact of IR on atherosclerosis. Conversely, in those with these conditions, other mechanisms, such as endothelial damage, endothelial dysfunction, inflammation, and oxidative stress caused by high blood pressure, combined with lipid deposition, and formation of fatty streaks due to dyslipidemia, may play a more significant role (28, 29) in evolution of CA, potentially attenuating the observed association with the TyG index.

Our study further demonstrated that the association between the TyG index and CA including plaques and stenosis remained statistically significant in non-hyperglycemic individuals and overweight/obese individuals. TyG index has been reported to be useful for the early identification of patients without glucose metabolic disorders at high risk of developing CVD (30). Overweight and obesity lead to dysfunction of adipocytes, which primarily produce and secrete pro-inflammatory adipokines. These adipokines participate in regulating inflammation, energy expenditure, lipid metabolism, and endothelial dysfunction in the body (31). This dysfunction increases the likelihood of insulin resistance, manifested as an elevated TyG index, which can trigger atherosclerosis. It indicated that the TyG index is still an important indicator for assessing CA, even in individuals with non-hyperglycemic and overweight/obese individuals. The absence of association with CA including plaques in hyperglycemic individuals may due to the limited power for its small event counts. In future, we need to further expand the sample size for thorough investigation. Furthermore, this association with CA including plaques was not observed in normal/lean-weight individuals. This is consistent with Zeyu Liu *et al's study* in the full-adjusted model (32).

Previous studies confirmed a linear dose-response relationship between the TyG index and carotid plaque incidence (33). Our study was consistent with previous studies. Furthermore, our study revealed that individuals aged ≥49 years have a higher probability of developing CA compared to those aged <49 years, all with a linear dose-response relationship. This finding underscored the fact that arteriosclerosis progressively develops with advancing age. Sex-specific analysis showed a linear TyG-CA association in both sexes, but males had a higher CA probability than females. This results are consistent with the findings of numerous studies (3). Also, TyG and CA showed a positive linear relationship in the groups with or without hypertension, hyperglycemia and dyslipidemia, respectively. Individuals with metabolic abnormalities such as hypertension, hyperglycemia or dyslipidemia had a higher CA probability than those without metabolic abnormalities. It is well established that metabolic abnormalities are a risk factor for CA (3). Metabolic abnormalities, including hypertension, hyperglycemia, and dyslipidemia, amplified the TyG-CA relationship, with affected individuals showing higher CA risk at the same TyG index level. Different BMI stratification analysis revealed a positive linear TyG-CA association in overweight/obese participants and a nonlinear one in lean/normal-weight participants, suggesting BMI influences this relationship via distinct mechanisms.

A study from the NHANES database shows that a U-shaped association between the baseline TyG index with all-cause and cardiovascular disease (CVD) mortality in CVD patients with diabetes or pre-diabetes. Threshold of the baseline TyG index values are 9.05 in all-cause mortality and 8.84 in CVD mortality (34). Our study identified a U-shaped association between the TyG index and CA in lean/normal-weight individuals, with a significant threshold effect at TyG = 8.112. We found that each unit increase in the TyG index reduced CA risk by 74% when below this threshold, while exceeding it increased the risk by 65%. Its potential physiological rationales may be adipose tissue insulin sensitivity thresholds and adipose tissue expandability limits in lean individuals (35). Other evidence suggests that extremely low levels of triglycerides (TG) or fasting plasma glucose (FPG) can have detrimental effects on health and may contribute to disease development (36). Hypoglycemia has been shown to elevate counter-regulatory hormones such as adrenaline, which can lead to vasoconstriction and increased platelet aggregation, thereby raising the risk of cardiovascular or cerebrovascular events (37). Therefore, it is crucial to maintain an optimal TyG index, as both excessively high and low levels can lead to adverse health outcomes.

### 4.1 Study limitations

#### 4.1.1 Causality and temporality

It should be noted that the study design was retrospective and observational, thereby precluding the establishment of definitive causal relationships and creating a gap in understanding dynamic changes over time due to the lack of longitudinal data on TyG and CA trajectories. Future research should prioritize longitudinal or Mendelian randomization studies to infer its causal relationship.

#### 4.1.2 Sample size

As a single-center study with a limited sample size, potential data bias might persist due to residual confounding factors, despite the use of multivariate adjustment and subgroup analyses. Furthermore, small sample sizes in certain subgroups (e.g., stenosis >50%, hyperglycemic participants) may have reduced statistical power. Larger multi-center cohorts are necessary to validate our findings.

#### 4.1.3 Limitations of confounding factor adjustment

Although we have made efforts to control for known confounders, there may still be important variables that are overlooked, which could affect the interpretation of the results. Future interview surveys should be more comprehensive, including not only medical history but also detailed information on medication use and lifestyle factors such as diet, exercise, sleep, and mental health. This will help to better understand how these factors influence the study outcomes and improve the validity and clinical relevance of the research.

## 5 Conclusion

This study demonstrated a significant positive association between the TyG index and the risk of CA, particularly with carotid plaques and stenosis, across a large population aged 18 years and older. Subgroup analyses revealed that the TyG-CA association was stronger in younger individuals, males, and those without hypertension, dyslipidemia, or hyperglycemia, highlighting the modifying effects of age, sex, and metabolic conditions. A U-shaped relationship was observed in lean/normal-weight individuals, with a threshold effect at TyG = 8.112. While the TyG index shows promise for CA risk stratification, its clinical implementation should account for sex-specific inflammatory responses and oscillatory lipid patterns (38). The TyG index may complement existing tools, but further prospective validation is needed to assess its incremental value. Combining TyG with measures of neutrophil activation or gut microbiota composition may improve early detection of high-risk phenotypes.

## Data Availability

The original contributions presented in the study are included in the article/Supplementary Material, further inquiries can be directed to the corresponding authors.
