# Supplementary material for: A retrospective cross-sectional study of association between triglyceride-glucose index and carotid atherosclerosis
Source: Front Cardiovasc Med. 2025 Jun 6;12:1611466. doi: 10.3389/fcvm.2025.1611466 (PMC12179170; doi:10.3389/fcvm.2025.1611466)
Supplement: Supplementary file 1 [file Supplementaryfile1.doc]

| Variables | Total  (n = 8600) | age<40  (n = 1676) | 40≤age<50  (n = 2756) | 50≤age<60  (n = 2452) | age≧60  (n = 1716) | *p* |
| --- | --- | --- | --- | --- | --- | --- |
| CA, n (%) |  |  |  |  |  | < 0.001 |
| 0 | 5256 (61.1) | 1557 (92.9) | 2147 (77.9) | 1180 (48.1) | 372 (21.7) |  |
| 1 | 3344 (38.9) | 119 (7.1) | 609 (22.1) | 1272 (51.9) | 1344 (78.3) |  |

**Table S1 Distribution of CA in Different Age**


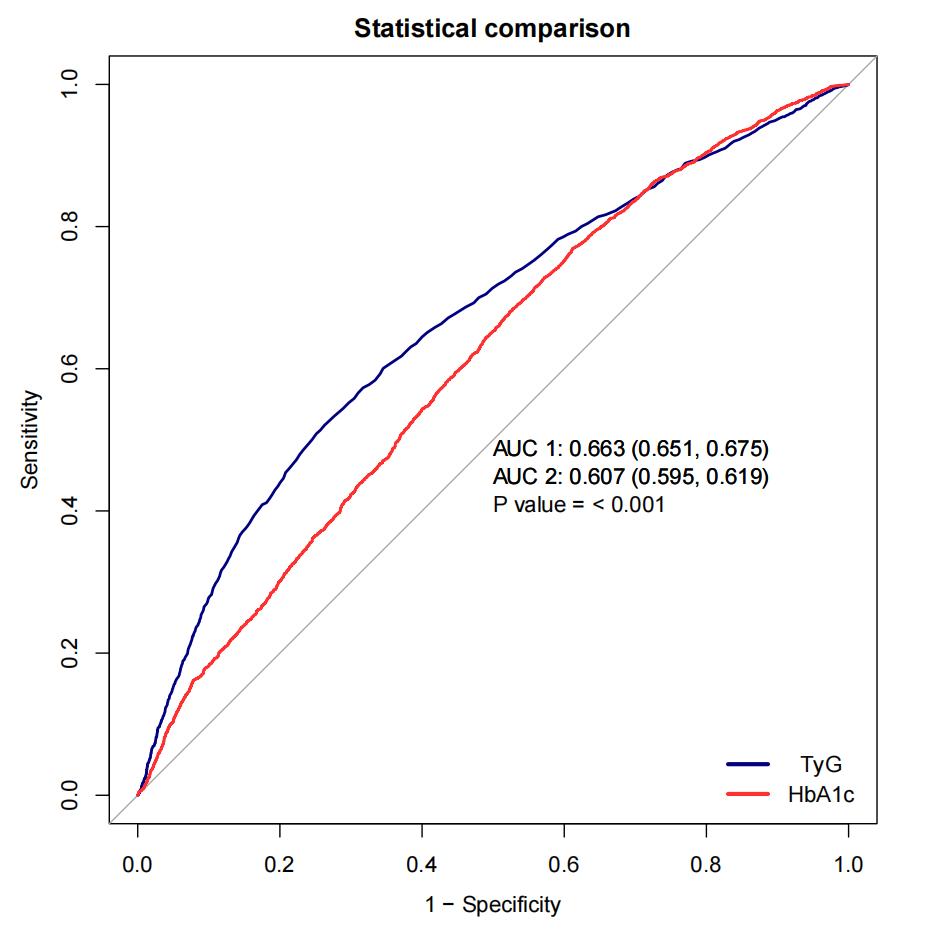


**Fig. S1 Comparison of the TyG and HbA1c.**


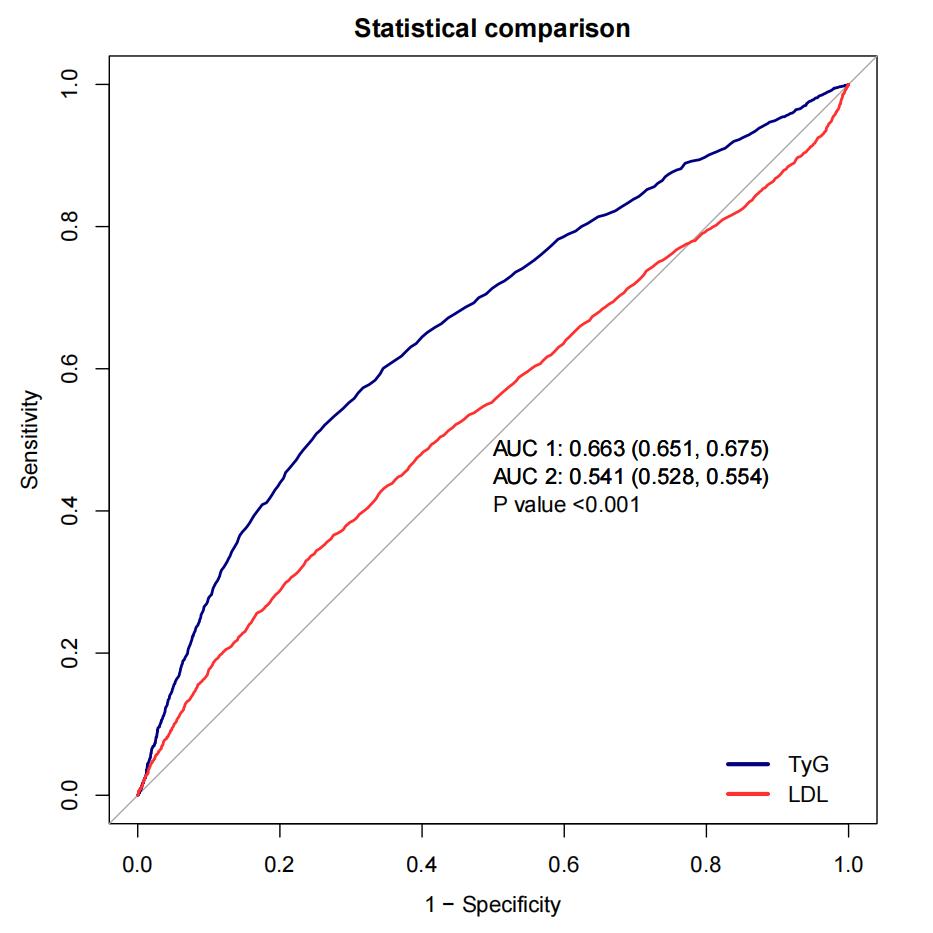


**Fig. S2 Comparison of the TyG and LDL.**
